# ‘What is Your Job?’: A Qualitative Analysis of the Deployment, Utilisation, and Contribution of Support Workers in Diagnostic Imaging Services in England

**DOI:** 10.1002/hpm.70005

**Published:** 2025-07-05

**Authors:** Sarah Etty, Beverly Snaith, Robert Appleyard, Julie Nightingale

**Affiliations:** ^1^ Sheffield Hallam University Sheffield UK; ^2^ University of Bradford Bradford UK; ^3^ Mid Yorkshire Teaching NHS Trust Wakefield UK

**Keywords:** allied health, deployment, skill mix, support workers, workforce

## Abstract

Support workers (SWs) form the largest section of the NHS workforce, and the ongoing NHS workforce crisis underscores the need for their efficient utilisation. This study explored the deployment of imaging SWs within NHS radiology departments in England, forming part of a larger multiphase research project funded by the National Institute for Health Research (NIHR). It involved multi‐centre case studies across nine radiology departments, employing a thematic analysis of focus groups and interviews with a range of radiology staff, including SWs themselves (*n* = 113). Results showed that recruitment of SWs was generally not challenging, however, retention was variable potentially due to limited opportunities for career progression and a lack of role understanding. Deployment strategies varied significantly across sites, which sometimes influenced SW effectiveness and were often selected for service need rather than SW development. Role scope was often unclear and training inconsistent which may exacerbate poor role understanding, and the lack of clear career pathways outside of professional registration conflicted with SWs' strong desire for progression. SWs are highly valued, crucial to operational efficiency and excellent patient care. Efficient deployment of SWs within NHS radiology services is crucial for alleviating workforce shortages and improving service delivery, however, this is impeded by the variability in role definition and deployment practices evidenced in this study. Standardising role titles, responsibilities, and training, and creating clear progression pathways could help to fully harness the capabilities of SWs in healthcare settings. National frameworks offer recommendations for standardisation, but this study suggests implementation remains inconsistent or delayed.

## Summary

- There are three models of deployment for Support Workers in imaging departments
- Support Workers are a highly valued but underutilised workforce
- Variation and lack of progression opportunities are hindering their potential
- More research is needed on Support Worker deployment across other professions

## 1 Introduction

In the United Kingdom (UK), the National Health Service (NHS) has been facing an ongoing workforce crisis. Between March and June 2023 there were reported to be over 125,000 vacancies [1]. Clinical support staff are the largest staff group within the NHS workforce [2], with their numbers increasing by 40% from 2010 to 2023. However, the NHS Long Term Workforce Plan estimates that ‘more than 204,000 new support workers will be required to meet demand over the next 15 years’ [3](p17). Support workers (SWs) are deployed across many healthcare settings [4], with varied roles including both patient‐facing [5, 6] and clerical functions [6].

SWs are essential to healthcare service delivery, and have been described as ‘the backbone of the NHS’ [5](p18). The 2013 Cavendish Review [5] and subsequent report [7] identified that SWs are frequently underutilised, undervalued, inconsistently deployed, and often unable to progress their careers. Despite this concerning finding, surprisingly few research studies have explored the deployment of this workforce [8]. An international scoping review identified that SWs across the allied health professions (AHPs) were underutilised, and that registered practitioners were reluctant to delegate to this staff group due to a lack of clarity regarding their scope of practice [8]. Inconsistency in deployment was also highlighted, and a need for further research to identify how SWs can be optimally utilised. This inconsistency even extends to their identity, with a report on maternity SWs identifying 22 different job titles [9].

The NHS workforce crisis is particularly acute within diagnostic imaging services (radiology) with a national shortage of consultant radiologists and radiographers, [10, 11] which directly impacts on how long patients wait for scans and results. In response to this, three high profile reports emphasised an urgent need to develop the capacity and capability of unregistered support staff [12, 13, 14]. Within the imaging support workforce, a three‐tiered structure exists with each tier corresponding to a pay band in the 'Agenda for Change' system used by the NHS for employee pay [5, 15]. This includes: (1) Clinical Support Workers (band 2); (2) Senior Clinical Support Workers (band 3); (3) Assistant Practitioners (band 4), in ascending order of autonomy and responsibility [5, 15]. The key differences between a SW and a registered AHP (in this context diagnostic radiographers) are the level of professional training and qualifications, scope of practice, and autonomy, and registration with a regulatory body appropriate to their profession. While autonomy increases with the three‐tiered support workforce structure, SWs work under the supervision of a registered AHP, whereas AHPs are able to work entirely autonomously. Therefore, with appropriate supervision, imaging SWs can undertake patient‐facing activities that were formerly the domain of registered staff [14]. This can then release those staff to undertake more advanced tasks, such as radiographers undertaking complex imaging procedures or reporting, thereby releasing medical staff time. However, effective implementation of SW skill‐mix is likely to be challenging due to a lack of understanding of best practice. A scoping review on SWs and assistant practitioners (APs) in UK imaging services confirmed a lack of research, finding only one article that explored SW deployment, solely from the perspective of managers [16].

Increasing the overall numbers of staff within the NHS may contribute towards alleviating the workforce crisis. However, effective deployment of all staff groups is essential to maximise their potential, particularly the opportunities afforded by appropriate utilisation of the support workforce. The aim of this article is to investigate the ways in which SWs are utilised within imaging departments in England, and the contextual factors that influence their deployment, from the perspectives of different staff groups, and importantly including SWs themselves.

## 2 Method

This study forms part of a larger multiphase research project funded by the National Institute for Health Research (NIHR ‐ NIHR133813) which investigated the development, deployment, and contribution of the Support and Assistant workforce to diagnostic imaging activity across England to determine effective models of practice. Following ethical approval and NHS site permissions [22/HRA/4272], this phase of the project employed a multi‐centre qualitative case study approach following guidance by Stake [17], and Crowe et al. [18] This study recruited nine different NHS imaging departments, selected using an evidence‐based approach from a larger cohort of 24 NHS Trusts in England that formed an earlier phase of the project [19]. Drawing on data regarding the proportion of SWs and APs employed in imaging services [20], three sites from each of the following categories were included: ‘high’ (departments with a median SW proportion of approximately 30%), ‘medium’ (approximately 20%) and ‘low’ (approximately 10%). Sites were selected to represent different geographical regions, settings and organisational types. A full account of the site and participant recruitment has been reported in another article which reviewed the primary cross‐case outcomes [21]. The current article reports the findings specific to SWs (bands 2 and 3).

The imaging support workforce and senior staff with management of SWs and APs were invited to participate at each case study site, employing a convenience sampling approach. Data were collected through interviews with senior staff such as managers and Speciality Leads (SL), and focus groups with SWs and APs facilitated by two members of the research team. In total, 39 interviews and 15 focus groups were completed, with an overall sample size of *N* = 113 (see Table 1). The majority were completed in person, but due to the demands on services and availability of staff, a number were completed online (interviews *n* = 13, focus groups *n* = 1). Topic guides for interviews and focus groups are included in the Supporting Information S1: Appendices (1–3). To ensure participant anonymity, no demographic data was collected.

**TABLE 1:** Summary of sites visited.

Site	Setting	Participants	SW full time equivalent (FTE)	
		*N* = 113	Actual establishment as reported from trusts	Figures reported in ESR (electronic staff record) data
1 (H)	Medium size service, coastal. 1 main site and further community sites	1 SW focus group (*n* = 4)	29.2	23.73
		1 Trainee AP focus group (*n* = 5)		
		1 interview with AP (*n* = 1)		
		3 interviews with SLs (*n* = 4)		
2 (H)	Large size service, coastal. 1 main site and a further satellite site	2 SW focus groups (*n* = 8)	27.97	39.31
		1 focus group, and 1 interview with apprentice radiographers/APs (*n* = 3)		
		5 interviews with SLs (*n* = 5)		
3 (H)	Medium sized service, city/rural. 1 main site and further community sites	1 SW focus group (*n* = 7)	46.8	68.44
		1 AP focus group (*n* = 5)		
		4 interviews with SLs (*n* = 4)		
4 (M)	Small sized service, city. 1 main site and further community sites	1 SW focus group (*n* = 7)	39.34	15.35
		2 interviews with managerial staff (*n* = 2)		
		2 interviews with SLs (*n* = 2)		
5 (M)	Large sized service, city. 2 main sites and 2 community sites	3 interviews with SWs (*n* = 3)	28.96	61.84
		1 interview with an AP (*n* = 1)		
		3 interviews with SLs (*n* = 3)		
6 (M)	Large sized service, city. 2 main sites and a further community site	1 SW focus group (*n* = 5)	Not available	33.41
		1 AP/trainee AP focus group (*n* = 7)		
		1 interview with managerial staff (*n* = 1)		
		1 interview with SL (*n* = 1)		
7 (L)	Large sized service, city. 1 main site and 4 satellite sites	1 SW focus group (*n* = 10)	26.83	19.36
		1 AP focus group (*n* = 6)		
		2 interviews with managerial staff (*n* = 2)		
		3 interviews with SLs (*n* = 3)		
8 (L)	Medium sized service, coastal. 1 main site and 3 satellite sites	1 SW focus group (*n* = 2)	24.48	1.33
		1 AP/trainee AP focus group (*n* = 2)		
		3 interviews with SLs (*n* = 3)		
9 (L)	Medium sized service, coastal/rural. 1 main site.	1 SW focus group (*n* = 3)	Not available	3.8
		4 interviews with SLs (*n* = 4)		

Abbreviations: H, high SW proportion; L, low SW proportion; M, medium SW proportion.

All interview and focus group transcripts were analysed thematically by at least two researchers, one a registered radiographer and the other a psychologist with former support worker experience, providing both emic and etic perspectives. Analysis was supported by Quirkos software [22] and regular team debriefs to agree on emerging themes and categories and following guidance as described by Braun and Clarke [23]. Data saturation was not used as a criterion for determining sample size. The nine case study sites, pre‐selected to reflect a range of different deployment models, were expected to each provide important but potentially unique insights. Including all nine sites, and hearing all relevant voices within each site, was valued as important for transferability of the results.

## 3 Results

Table 1 shows the characteristics, participants, and numbers of SWs for each site. This section will report the findings of the analysis relating to the following key themes: Identity, Recruitment and Retention, Deployment, Role scope, Utilisation, Training and Education, Career Pathway, Role awareness, and Impact.

### 3.1 Identity

Job titles varied widely, with the majority referring to SWs as Radiology Department Assistants, shortened to RDAs, but others using the generic term Health Care Assistant (HCA) (Table 2). For one trust job titles even differed between their two hospital sites. Additionally, one trust still used the outdated job title of ‘Helper’, which was not appreciated by SWs.

on my payslip [it states] support worker/helper, as if I’ve just come for the day, I’ve volunteered for the day.

SW—site 1

**TABLE 2:** SW Job titles used at each site.

Site	Job title
1	Support worker/helper
2	Radiographic department assistant
3	Radiology department assistant
4	Health care assistant/medical imaging assistant/radiology care assistant
5	Radiographic department assistant/health care assistant
6	Imaging assistant
7	Health care assistant/radiology assistant
8	Radiology support workers/clinical assistant
9	Support worker

Uniforms were often standardised across hospital services, so band 3 imaging SWs would wear the same uniform as the HCAs working on the wards.

It’s standardised. Yeah. It’s not denoted by department.

Manager—site 4

### 3.2 Recruitment and Retention

The SW workforce were mostly female with a broad age range, with managers reporting that SWs tended to reflect the ethnic diversity of the region.

They all are local, so there’s not a huge ethnicity mix.

SL—site 9

The majority of trusts had no difficulty in recruiting SWs, however one trust cited their main difficulty as not having enough funding to employ sufficient SWs.

We don’t tend to have an issue recruiting. Whenever posts go out, they always seem to do fairly well and I think we get quite a few applicants.

SL—site 9

SWs were sometimes recruited from elsewhere within the trust, or from other roles aligned to imaging.

I’d worked for 10 years in radiology as admin, … I love working there, I wanted to work more with patients. … I saw the job advertised as a support worker in radiology [diagnostic imaging], and I thought that was the perfect job for me, so I applied.

SW—site 9

Many of the SWs recruited in more recent years have higher education qualifications (degrees) and use the SW role to gain experience in healthcare and explore opportunities for progression.

quite a lot of them [SWs] have degrees … They’re not sure what they want for their future and sort of initially take the job so they can come and find out more about radiography and whether or not they want to then pursue a career in radiography.

SL—site 6

Retention of SWs was not seen as a problem for some sites, with some SWs working in the imaging department for 25 years or more.

I think it’s the most stable workforce group we’ve got, to be fair.

SL—site 3

However, some managers reported that retention was challenging, sometimes linked to career progression opportunities.

I have almost an annual turnover of healthcare assistants.

SL—site 7

it’s the younger ones that we tend to get that do move on, but it’s good that they’re moving on because most of them are moving on into some sort of professional career.

Manager—site 4

However, SWs often cited other reasons for high turnover, such as a lack of opportunities to progress, or roles not being as described.

I know a lot of people at the moment looking for jobs elsewhere, because there is the lack of support … There’s no progression, and people want to progress.

SW—site 8

Yeah, there was one not long ago came, she was like I didn’t think it was going to be like this, I’m not going to stay, handed her notice in on the first day.

SW—site 1

There were other aspects of the SW role that caused dissatisfaction, such as low pay and a predominance of administrative tasks.

We’ve compared it with what the likes of somebody from Asda [a supermarket chain] gets paid. They get paid more than us.

SW—site 1

We’ve got to do everything on reception… If I wanted to work in reception as a ward clerk, or whatever they’re called, I would have applied. We’ve already lost a colleague because of it.

SW—site 2

Despite there being elements of the job that some were dissatisfied with, when asked if SWs enjoyed their job, the answer was unanimously yes.

I wished I’d done it years ago, if I’m honest, I’ve really enjoyed it, never dread coming into work.

SW—site 4

This also appeared to be influenced by their department culture, with many remarking on the friendly atmosphere and that they felt part of the wider team.

It feels like we’re not just band 3s, we’re all equal in our department. … nothing’s below a radiographer to do, and nothing’s above a clinical support worker.

SW—site 8

Two sites had set up a regular meeting for SWs, and at one site this included naming a member of the workforce as ‘Support Worker of the month’.

[We set up a] RDA meeting, and then at the meeting we’ve got RDA of the month, we’ve also got now static RDA of the month, and they’ll get a certificate and a little trophy.

SW—site 3

These meetings were considered helpful in creating a community, aiding information sharing, and giving SWs the opportunity to voice any concerns and feel listened to

So we have them in the RDA focus groups, it’s just really good because we bring up anything that anyone’s got any comments, or they want to discuss anything. We’ll talk about things, and just try and make the role easier.

SW—site 3

### 3.3 Deployment

Deployment varied, with two main approaches; a rotational model where SWs worked across different imaging specialities, and others using a model where SWs were static within a single speciality area. These models even differed within one site.

I think at [Hospital A] they’re CT [Computed Tomography] and MR [Magnetic Resonance], then X‐ray and ultrasound. Whereas … at [Hospital B] it’s CT and MR, then ultrasound, and then X‐ray, so we’ve got three different pools of assistants.

SL—site 2

Some hospitals also employed a hybrid of the two, with some SWs being described as ‘*static*’, and others ‘*rotating*’. There were mixed opinions on how well each model of deployment worked. Managers often preferred a rotational staff model, as this addressed the problem of covering staff absence, whereas some SLs and SWs preferred static deployment. Some SWs struggled with some of the specialities, particularly if significant time had passed since last working in that area.

I’m quite supportive of having the radiology support workers on my team, rather than having a rotational amount of staff … it gives us opportunities to focus their training on certain areas, give them opportunities for career development, that kind of thing.

SL—site 4

We have got a big team, so that often happens that you might not go to a particular area for weeks on end, so it’s challenging.

SW—site 3

One site had recently changed their model of deployment from rotational to static, which staff felt had been a positive change.

they seem much more settled, they know what they’re doing, they take more responsibility … They tend to have taken much more pride and being more proactive in how they work as a team, they work as a team a lot better…

SL—site 1

SWs were utilised within most specialist imaging areas, however, x‐ray was the area in which they were least visible.

We do have some support workers working in fluoro [fluoroscopy], not so much in X‐ray.

Practice Educator—site 7

Differences were also apparent between specialities. Ultrasound was often cited as having a slower pace during which SWs would spend more time with patients, whereas CT was described as an extremely fast‐paced environment in which multitasking and prioritisation skills were essential. It was also generally expected that within cross‐sectional imaging (CT and MR/MRI) SWs would be proficient in intravenous (IV) cannulation.

It’s a lot more stressful in CT. There’s a lot more happening and it’s a lot more manic, so not everybody can actually cope with that.

SW—site 1

CT was reported to be so busy that one SW was unable to remember the last time they were able to attend a meeting, with another pointing out the unfairness of this, as they perceived radiographers as able to attend meetings when needed.

But then that seems a bit unfair sometimes, because if you have a band 6/7 [radiographer] in plain film [X‐ray] or wherever, they’d have blocked out meetings.

SW—site 3

The training requirements could differ depending on speciality, as some areas have more safety considerations than others.

the actual training for the RSWs [radiology SW] to come and work in MR is probably a bit more detailed than the other areas, because they have to do their MR safety training as well.

SL—Site 8

### 3.4 Role Scope

Variation seemed to exist between sites in the types of tasks that SWs completed as part of their role.

when you’re then working with other hospitals, you realise that they don’t use assistants [SWs] in quite the same way that we do.

SL—site 3

There was a consensus across speciality areas that the key SW role was to ensure a smooth patient flow through the department and assist radiographers where necessary.

ideally they should be prepping the equipment. So helping us to put the coils onto the table, restocking linen, things like that in the rooms, clinical supplies, they do a first pass safety check with the patients, so they run through the safety questionnaire initially. At that stage they’ll check their details. They’ll explain the examination to them. If they need cannulating, they’ll cannulate as well, get the patient changed and answer any queries that they’ve got, then they should hand that information over to the radiographers and then the radiographers carry on with that case.

SL—site 4

they’re managing all the workflow … then actually it just makes everything flow and everybody’s working to sort of the top of their game then.

SL—site 3

The main difference in scope of practice between band 2 and band 3 SWs was that band 3s took on more clinical tasks.

The band 3s, their primary part of their role is cannulation, but in addition to that, they are responsible for doing stock control … they can order a certain amount of stuff from pharmacy, contrast and everything’s ordered on a weekly basis…

SL—site 6

Some sites had either recently or were currently going through the process of re‐banding their SWs from band 2 to 3, and were updating job descriptions to more accurately reflect their roles.

I think there’s a few members that have been here quite a long time that feel that they work at a higher level … and with the re‐banding that’s going on at the minute, they’re now feeling uplifted.”

Manager—site 4

we are gradually trying to move away from band 2s so everybody will come in on the band 3 setting.

SL—site 7

In some sites SWs begin their employment at band 2 and then progress to band 3 upon the completion of a defined in‐house training programme. At another, that adopted the hybrid (rotational and static) model of deployment, SWs could apply to be static in a particular speciality, which could result in a promotion from band 2 to 3.

### 3.5 Utilisation

SWs often aspired to do more, a view that was echoed by managers.

I’m too old to go to uni, that’s why I think sometimes it would be nice to be able to just, those little bits which you’re more than capable of doing.

SW—site 3

I feel the radiographers take on a lot of duties which is below that of what a degree should be. I feel you could run a scanner much more efficiently with more RDAs and the radiographers do more of the radiographer role instead of getting on and off the table, that kind of thing. I feel here it’s underutilised.

SL—site 7

At one site, SWs felt the level of their underutilisation in one particular area was personally demeaning.

It is quite degrading, you just sit outside [the scan room], you almost get like your fingers clicked, then you come in and you clean, but only every 20 minutes or something.

SW—site 9

Additionally, at some sites SWs were often deployed flexibly across the whole imaging service, for example, to provide cover in administrative areas.

So they are trained to work on the desks as well and be able to cover the admin and clerical people. They also book appointments as well.

SL—site 3

Some sites had specialist paediatric SW roles for which they had received extra training, but not funding. Other further training included awareness courses for conditions such as dementia, autism, hearing impairment etc.

We do have a wide variety of patients who turn up, from autism to learning difficulties, all sorts. You get training, specific training on how to help those patients, our goal is to help these patients have their scan.

SW—site 9

One site had trained their SWs in point of care testing (PoCT) of blood samples as a supplement to their IV cannulation skills and a few sites had been discussing the potential for SWs to train to complete ultrasound guided cannulation for difficult access. Further, another had trained one of their SWs in administrative cancer tracking for ultrasound.

I’d love to do more, but there’s talks of ultrasound guided cannulation, and I love stuff like that.

SW—site 9

They [SWs] assist nurses with biopsies. And they’re about to do the, what we call the PoCT, which is taking blood samples for eGFR and everything.

SL—site 6

There were a small number of instances of SWs being asked to complete tasks outside of their job description, with varying degrees of enthusiasm from the support staff.

they wanted us to sign off an MRI as RDAs [Radiology Department Assistants] for the safety questionnaire and we all kicked up a fuss saying, well no, because we’re not professionals, we’re not registered, so we’re not signing off.

SW—site 3

they have been trying for some time to get us to do ultrasound [guided] cannulation, which is something that the doctors normally do.

SW—site 2

SWs worked a variety of shift patterns as appropriate to the modality in which they were working. Often SWs would work long days in MRI and CT, but with shorter days in other areas such as ultrasound, in line with radiographer rosters.

Many sites had a separate pool of SWs to cover out of hours shifts, however this was not universal.

They [SWs] work weekends, but we don’t have them in nights.

SL—site 9

The management of SWs differed between sites, with the model of deployment being the main determinant. If the site employed a static deployment model they were often managed by the speciality lead radiographer within their designated area of work, whereas for rotational staff someone from the senior radiography team took on the responsibility for coordinating the entire SW rota. However, in one case SWs were managed by the radiology lead nurse, which was seen by SWs as positive, but it was also felt that there was room for a SW to take on some managerial responsibilities, a view echoed by a manager on another site.

it would be nice if there was a senior or a lead support worker within a department that could take a bit of the clerical pressures off the management, but also be there for your team as well as like a go‐between.

SW—site 9

It would be ideal if we could have somebody just a grade above them [SWs].

SL—site 6

Two sites did employ a lead SW with some managerial responsibilities at band 4, however, these roles differed between sites. At one site they had a team of 3 SWs that managed all other SWs, taking responsibility for their annual leave and sickness. At the other site the single SW lead managed only the SWs based in ultrasound.

…their overall management would sit with the RDA lead so that’s where their annual leave would be agreed, where they would have any personnel discussions, sickness, return to work type things.

SL—site 3

…there is an RDA lead and they come to the managers' meeting but they only lead probably a third of the RDAs in the department, so it is a bit odd that.

SL—site 2

### 3.6 Training and Education

SW training differed between sites. Many sites had defined SW competencies, but training differed from informal processes to formalised programmes, extending from 6 weeks to 6 months, influenced by their scope and range of specialities covered. All imaging specific training was ‘in‐house’ although a number of organisations also expected the completion of the Care Certificate [24].

SWs were most often mentored by other, more experienced colleagues.

the first four weeks we’d be partnered up with someone that’s more experienced.

SW—site 7

However, in some cases SWs were thrown into their roles with little in the way of support, or felt that there was no continuity in their training when they started in their roles.

I sat down on the computer today and then this girl sat next to me, and then one of the band 7s [radiographers] came in and went, ‘she just started yesterday are you all right just to look after her? … I haven’t got time to show you anything at the minute’ and I felt a bit sorry for her.

SW—site 1

When I started six months ago the first week it was a different person every single day … I was being told different things.

SW—site 4

The sites had a formal progression pathway requiring achievement of specific competencies to enable movement from band 2 to band 3.

we start people at band 2 and we’ve got competencies that they work towards and once they achieve those competencies we re‐band them to 3.

SL—site 2

### 3.7 Career Pathway

Across all sites there was evidence of SWs' desire to progress in some way, either within their current role, or in a new direction. Some were happy with their role and did not wish to pursue progression outside of this, whereas others were clear in their ambition to train to be an AP or registered radiographer.

Age was often a factor in whether SWs were hoping to progress further.

it’s usually the younger ones who want to sort of progress further.

SL—site 5

I think at my age I’m quite happy to carry on as I’m doing.

SW—site 3

Staff in some sites felt that for SWs in x‐ray there was very little opportunity for progression, particularly when compared with the cannulation training available to SWs in CT and MRI.

Only one site was using apprenticeships, and one SW felt that this had been the only way for them to have gained employment within the department as they had struggled with education previously.

I don’t think I had enough qualifications to be a band 2 straightaway. So it helped me get into that. … at the time I didn’t have my maths.

SW—site 3

The opportunity to progress was often cited as a reason for applying for SW roles within imaging.

I only came, took the support worker role because it was mentioned in the interview. I’d researched it and that would be the next progression, so that’s the only reason I actually applied to be a support worker for this role.

Trainee AP—site 1

RDAs come in as band 2, so then there’s that progression to become a band 3 when band 3 positions come up. A lot of our RDAs do go on to train as APs or do the radiography degree. For RDAs it’s the step up to band 3, rotational or band 3 static, but then that is sort of the limit.

SW—site 3

There were differing opinions on the availability of progression opportunities for SWs, with some SWs feeling that these opportunities never appear, and some senior registered staff not being aware of the scarcity of such opportunities.

I don’t think there’s a lot to support us really, because they keep saying, oh there might be something in the future. They keep saying that.

SW—site 8

The sky is the limit for most of them on what they want to do.

SL—site 7

Financial constraints were often cited as a reason for a lack of progression opportunities for staff, including registered practitioners. One speciality lead stated that if opportunities for progression are unavailable for the higher bands, this has a knock‐on effect on progression opportunities for the lower bands.

Again, finance restricts us… I would love to have more reporting radiographers … I’ve asked every year I’ve been in post for business planning for band 7 posts to train reporting radiographers, and the answer has been no. So, unless you push that top end, you can’t bring people up from the bottom.

SL—site 3

### 3.8 Role Awareness

Some SWs felt they did not know what to expect from the role prior to employment. SWs also mentioned a lack of understanding of their roles from other staff groups, such as health care assistants (HCAs) from wards, managers, and even registered staff in some cases.

I think a lot of the HCAs off the ward come down with the patients, actually see it as an easy department. … We’ve heard them in recovery saying, diagnostic, they don’t do much down there, they just sit down. … but we have had some that have come down here and they haven’t lasted long.

SW—site 4

They don’t understand what it is you’re doing. They think you’re just standing there in the corner chaperoning.

SW—site 1

Some SWs felt that it was not made clear what the role actually involved.

I don’t think that that’s really explained at interview quite how clinical it is.

SW—site 2

### 3.9 Impact

SWs' absence was cited to have a big impact on the running of the department as they were reported to be essential to patient flow in the department.

if we didn’t have them, it would really impact everything that we do, every list that we run would be really impacted so yeah, they’re of a great value.

SL—site 4

if the consultants came round they won’t even start the list until there’s a support worker.

SW—site 1

What they do provide me with is that kind of comfort and scene setting for patients and they improve the efficiency through department. Because we have had times where we’ve had a radiologist had to run their own list and they just, they markedly go behind because they’re not thinking what’s next, what’s coming up, they don’t have that frame of mind to do that.

SL—site 7

Some SWs felt that their recent regrading to band 3 was recognition for the work that they contribute. It was also evident that registered staff greatly valued SWs.

I would say most shifts at the end of the day I do get a thank you from the radiographers or sonographer, which is quite nice.

SW—site 3

I can’t imagine us running our department without the support workers, basically. They’re an integral part.

SL—site 9

It was reported that SWs were also highly valued by patients as integral to ensuring patient wellbeing.

they love the support workers. They’re always very appreciative of having them because … they build up that close connection from the very beginning.

SL—site 3

It really makes a difference, especially if we’re having like an agitated patient, or patient is very anxious. Then at least the HCA is there to ease that patient, pacify that patient, it makes a difference. Even just to hold their hand while the doctor is doing biopsy, it really calms them down.

SW—site 7

for example today, we don’t have an RDA because of sickness over here. I think it affects the patient experience

SL—site 2

## 4 Discussion

This study has provided the first comprehensive investigation of the deployment of imaging SWs and includes the perspectives of this workforce, in addition to their registered colleagues. The principal finding from this analysis is variation; there is little consistency regarding role titles, deployment, training, or career progression opportunities.

**FIGURE 1:**
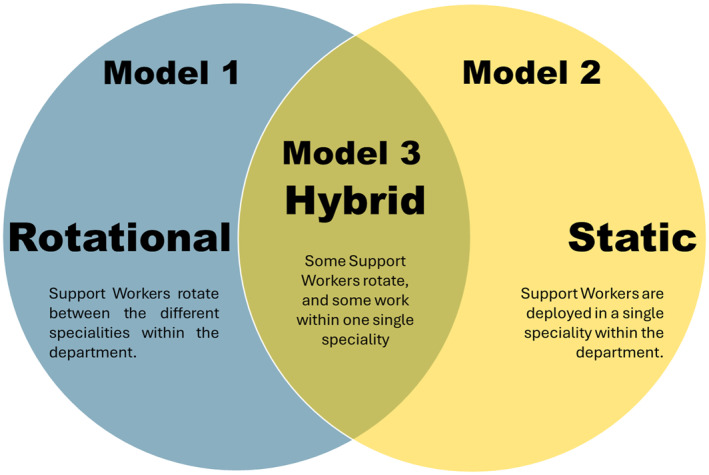
Models of SW deployment used across sites.

The most pivotal support workforce deployment decision was whether imaging services employed a rotational, static (single speciality), or hybrid deployment model (see Figure 1). Some SWs preferred static deployment in a specialist area that suited their personality or way of working, and focussing in one area allowed them to become experts. Others reported that they like the variety that rotational deployment provides, and managers appreciated the flexibility that this model offers in being able to keep a department running and as a way of enabling inevitable ‘firefighting’. Crucially, rotational deployment was driven by the benefits to service delivery, rather than the benefits it could provide to SWs. The hybrid approach utilised by some sites seemed to offer a suitable compromise, enabling progression opportunities for some to specialise, supporting the diversity associated with rotation for others, and allowing the flexibility needed to cover staff absence. An Australian study by Pinson et al. [25] showed that medical imaging assistants worked across four different areas within their departments, rather than working statically, however there was no discussion of the advantages or disadvantages to this model of deployment. Their study demonstrated that when using this rotational model, the majority of assistant time was spent in CT, suggesting that the complexity of work in this area requires more support than others, which could present an opportunity for progression. Indeed, SWs in this study highlighted the stark difference between CT and other areas in diagnostic imaging, including the fast‐paced nature of the work and the advanced skills associated with this area (cannulation, prioritisation, etc.). X‐ray was highlighted in Pinson et al.’s study as the area in which assistants spent the least time [25], a finding echoed in this study, suggesting that this area of diagnostic imaging is not a viable area for further development of the support workforce outside of training to be an AP.

Band 3 roles were reported to be more clinically focussed than band 2 roles, which were reported to be more aligned with patient flow. This differentiation between band 2 and 3 SW roles is important information not yet documented in previous research. A previous phase of this project [20] showed that some departments were opting to deploy either only band 2 SWs, or only band 3 SWs. In this phase of the project some sites were re‐banding their SWs to band 3, suggesting that managers are critically evaluating the tasks their SWs are undertaking and ensuring that they are meeting the changing needs of the service. There was also evidence of SWs being expected to complete tasks outside of their scope of practice, known as ‘role creep’ [26], with SWs in some cases refusing to undertake these tasks. This raises a question of the need for both roles. Band 2 roles are reported in the relevant guidance [14] to be entry level positions and were found in this study to be essential to workflow, and band 3 to be advanced roles with more clinical tasks. Arguably, both roles serve a valuable purpose, most notably as being the doorway to healthcare careers, and to make room for progression to more clinical responsibilities. The inclusion of both is likely to provide the stable entryway that enables ease of recruitment through band 2 roles and steady retention of the workforce through opportunities for progression. The removal of band 2 roles may cut off a valuable pipeline for recruitment into the support workforce, whereas the absence of band 3 roles would disrupt the career pathway through support workforce and registered practice roles.

Job titles varied widely for SWs, consistent with earlier phases of this wider project, [19, 20]. This has also been found to be the case for SWs in other fields, such as maternity [9]. One study exploring HCA roles found 37 different role titles across 6 different countries [27]. Additionally, the current study identified that some sites had all band 2 and 3 level clinical staff wearing the same uniform; rendering a ward‐based HCA indistinguishable from an imaging SW. This lack of identifiable connection to their departments, combined with a lack of standardised job titles leads to poor professional identity and visibility, a conclusion also highlighted in a review of the Cavendish report [7].

Training and education of SWs was inconsistent, with some sites adopting a thorough and comprehensive competency framework to complete upon initial employment, whereas others had a more informal approach exacerbated by inadequate staffing levels. Many sites required SWs to complete the Care Certificate [24] as a part of their induction if this had not already been completed. However, this is a generic health care course that is arguably more aligned to nursing than any allied health roles, so while this allows SWs to learn general care skills, it does not offer specific training in their area of work. Some sites had engaged with degree apprenticeship opportunities, but only one site was using the lower‐level apprenticeships to facilitate the recruitment of SWs. The lack of consistent, vocation‐specific training is an area of concern, and may contribute to the lack of progression opportunities within the imaging setting. Some sites were offering SWs the opportunity to receive patient‐focused education such as dementia or autism awareness training, potentially creating new opportunities for SWs to become champions for patients with these conditions within their departments, and something they could add to their curriculum vitae. However, opportunities to progress further within the SW role that was also reflected with a promotion to a higher banding were rare. The lack of opportunities for further training, the variation seen in SW activity, and the absence of clear, uniform frameworks for training and deployment will restrict the quality of work SWs are able to achieve, which is likely to substantially impact patient safety and satisfaction. A systematic review [28] showed that inadequate training was associated with high rates of burnout in HCAs. National SW frameworks [6, 14] have outlined important recommendations for this workforce, which, if implemented, would contribute to a more standardised, uniform application of SW roles. However, this study has shown that there are either barriers to fully implementing these recommendations, or a delay in the recommendations reaching the awareness of those that can instigate change. One avenue for progression was the SW lead role, implemented at only two sites within this study. However, the idea of SWs taking on more managerial responsibilities for their workforce was welcomed by registered staff, and in turn releases radiographer capacity. This progression pathway is not included in the frameworks for SWs in Allied Health [6] or Imaging [14] and the overall radiography workforce [13]. This suggests that this role is a relatively new innovation, but its introduction allows SWs to become leaders and progress without needing to move to registered status. This is important for recruitment and retention of this vital workforce. Seeing the support workforce only as the supply pipeline to the radiography profession may be inhibiting the implementation of expanded roles at SW level. This is equally as important to the registered workforce, as appropriately implementing skill mix in this way could improve retention of the registered workforce who were often tasked with providing time‐consuming staff management. Reducing this unnecessary workload would provide some of the backfill needed for enhanced and advanced practice.

Crucially, the opportunity to progress onto AP or radiographer roles was often cited as the reason SWs applied for their posts, however, they also reported that opportunities to actually complete this training were infrequent. This, coupled with the lack of opportunities for progression within the SW role, is likely to contribute to challenges in retention seen across this workforce.

SWs were appreciated by management and the registered workforce, however, some SWs felt that the registered workforce did not fully appreciate the extent of their contribution to the service provision, or how much they could benefit from appropriate support. This suggests that while the registered workforce highly value their support workforce, this is not being communicated effectively to them. It could also provide further evidence of lack of understanding in the registered workforce of the scope of SW roles. This could be addressed by providing better training for registered staff on delegation and supervision of the support workforce. Nevertheless, SWs were unanimous in their enjoyment of their jobs, and the sense of purpose and satisfaction they received from delivering high quality patient care. SWs compared the positive atmosphere and equal footing within the team to that of previous roles on wards which were often reported to be more hierarchical. These factors are arguably likely to be what keeps SWs in their roles in spite of the lack of progression and education opportunities.

This study reflects key insights similar to those found in previous studies examining the views of HCAs within UK GP (general practitioner) practices [29, 30] and extends to more recent assessments of Allied Health Assistants in Australia [31, 32, 33], and HCAs in physiotherapy roles [34]. Despite the variations in healthcare systems, there is notable consistency in experiences and perceptions, especially concerning the persistent issues of unclear role definitions and the lack of coherent and reliable career advancement opportunities. Further research on SW deployment and utilisation across all the Allied Health Professions, which is currently limited [8], could establish whether these similarities extend broadly across Allied Health. Additionally, measuring the impact of SWs within imaging services and across the other Allied Health Professions, and the benefits of different deployment models are much needed avenues for further research.

## 5 Conclusion

Despite being a highly valued workforce, opportunities for SWs to progress further either within their roles or into registered roles are infrequent, and service need is often prioritised over SW development. SWs lack clear professional identity, and their roles do not appear to be well understood by the registered workforce. Current SW frameworks are useful in outlining the possible career pathway to registered status but omits opportunities to develop *within* the SW role rather than outside of it, although some trusts are demonstrating innovative leadership opportunities. The tendency to view this workforce as the pipeline into registered roles when there are extremely limited occasions for this progression to occur is likely to be disheartening for SWs and may contribute to high turnover rates seen in some trusts. Widening the capacity for progression within SW roles allows development not just for SWs but also creates the space for registered staff to enhance their own practice and alleviates the pressures on this already overburdened and understaffed workforce. Further research is needed to examine whether these findings extend to further Allied Health Professions, and whether there is an optimal deployment model of the three identified.

## Conflicts of Interest

The authors declare no conflicts of interest.

## Supporting information

Supporting Information S1

## Data Availability

The data that support the findings of this study are available on request from the corresponding author. The data are not publicly available due to privacy or ethical restrictions.
