# Supplementary material for: ‘What is Your Job?’: A Qualitative Analysis of the Deployment, Utilisation, and Contribution of Support Workers in Diagnostic Imaging Services in England
Source: Int J Health Plann Manage. 2025 Jul 5;40(6):1220–31. doi: 10.1002/hpm.70005 (PMC12579519; doi:10.1002/hpm.70005)
Supplement: Supplementary file 1 — Supporting Information S1 [file HPM-40-1220-s001.docx]

## Appendix 1 – Modality Lead Interview Topic Guide

**Modality Leads – Interview topic guide (version 3)**

**Aims of the Interviews:**

To assess and explore:

1. The contribution of the S&A workforce to their specific modality
2. Supervision of the S&A workforce in the specific modality
3. Delegation of tasks/roles
4. Training and assessment of the S&A workforce (knowledge, skills, competence, use of technology etc)
5. Implications for safety
6. Implications for quality
7. Barriers and facilitators to deployment of S&A workforce in their specific modality
8. Concerns raised by patients related to care provided by the S&A workforce
9. Perspectives on the future role of the S&A workforce in their specific modality

**Interview schedule**

Main interview question items numbered. Follow up questions lettered underneath each main question

Introductions.

Address any queries about the study.

Clarify ‘rules’: All viewpoints are valid, avoid referring to names specifically, clarify that if names are inadvertently used then transcripts will be redacted to ensure anonymity.

Address SW and APs separately where necessary

At start of recording: This is <modality lead> at <site code>

1. What titles do you use for support workers and assistant practitioners?
   1. Is this a Trust title or a Radiology Services title?
   2. Are uniforms different to other staff?
2. Tell me about how support workers (bands 2/3) and assistant practitioners (band 4) are utilised in your modality area?
   1. What does their working week look like?
   2. Are rosters same, similar or different to Radiography staff (9-5, 24/7 etc)?
   3. How was SWs and APs scope of practice defined/developed in your area?
   4. What do they specifically contribute?
   5. Who manages SWs and APs? (organising rotas, PDRs recording sickness etc.)
   6. How are roles/tasks delegated?
   7. How are they supervised/supported in their role(s)?
   8. Impact on efficiency and quality of service?
3. What are the key facilitators and barriers to deployment of the support worker/assistant practitioner workforce? Why are the barriers there and how may they be overcome?
4. How are support workers and assistant practitioners trained in your modality area?
   1. In relation to developing knowledge, skills, competence, use of technology etc
   2. How are they assessed?
   3. What are your perspectives on how training and monitoring should be developed in the future?
5. Have there ever been any concerns over safety of service provision with regards to the use of support workers and assistant practitioners?
   1. If so how have they been overcome?
6. In your view, how do patients feel about being seen by/cared for by support workers and/or assistant practitioners?
   1. Have there ever been any concerns expressed? How have they been managed?
7. What are your perspectives on how support workers and assistants should be utilised in your modality area in the future?
   1. How far could you go with developing these roles? Are there any staff or management resistance to these developments?
8. Have the SW and Assistant roles impact on the development of Radiographer roles in any way?
   1. Explore impact on extended roles, advanced and consultant practice?
9. Finally, is there anything else you would like to convey that we haven’t already addressed?

## Appendix 2 – Service Lead Interview Topic Guide

**Radiology Service Lead – Interview topic guide (version 3)**

**Aims of the Interview:**

To assess and explore:

1. organisational structure of their unit
2. strategic approach to recruitment and deployment of S&A workforce
3. operational issues associated with 1 and 2 above
4. current and future deployment of S&A workforce in selected modalities (general, CT, MRI, US, Nuc Med etc as appropriate)
5. perspectives on optimal skills mix and workforce planning decisions with exploration of band 2/3 and band 4 deployment
6. barriers and facilitators to S&A workforce deployment

**Interview schedule**

Main interview question items numbered. Follow up questions lettered underneath each main question

Introductions.

Address any queries about the study.

Clarify ‘rules’: All viewpoints are valid, avoid referring to names specifically, clarify that if names are inadvertently used then transcripts will be redacted to ensure anonymity.

Explore SWs and APs separately where necessary.

At start of recording: “This is <service lead> interview at <site code>

I would like to begin by exploring how support workers (bands 2/3) and assistant practitioners (band 4) are utilised across imaging services in your centre?

1. What titles do you use for support workers and assistant practitioners?
   1. Is this a Trust title or a Radiology Services title?
   2. Are uniforms different to other staff?
2. What are the main operational issues associated with deployment of support workers and assistant practitioners across imaging services here?
   1. Who manages the SWs and APs? (PDRs, sickness, rotas etc)
   2. What drivers influence your deployment strategy?
3. How would you describe the strengths and limitations in how support workers and assistant practitioners are utilised in your centre? In other words, what works well and where is there need for potential development?
4. What are the key facilitators and barriers to deployment of the support worker/assistant practitioner workforce? Why are the barriers there and how may they be overcome?
5. Can you expand on SW and AP deployment across different modality areas. What works well? Where are there opportunities to develop?
   1. <site specific note> *May need to probe on specific modalities e.g. follow up on WS4 issues: apparently pivotal role of band 2s in CT, fewer support workers in general x-ray at N. Tees*.
6. What are your perspectives on the future role of the SW and AP workforce?
   1. How far could you go with developing these roles? Are there any staff or modality lead resistance to these developments?

Moving on to recruitment and retention of the support worker and assistant practitioner workforce.

1. How would you describe your overall recruitment strategy with respect to the support worker and assistant practitioner workforce here in?
   1. Do you recruit from other NHS roles in same organisation, directly from local community and non-NHS services, have links with educational institutions?
2. What are the main retention issues you face with respect to the support worker and assistant practitioner workforce here?
3. Tell me about the training and education of the support worker and assistant practitioner workforce here.
4. What works well and where are there specific challenges in advertising, recruiting, training and retaining the S&A workforce?
5. How have recruitment strategies changed over time?
6. In your view what works well in keeping the SW and AP workforce happy and motivated?
7. What plans do you have for developing recruitment strategies in the future?
8. Are there any initiatives to recruit from under-represented population groups? How successful are these?

Finally, is there anything else you would like to convey about the SW and AP workforce here in your Trust that we haven’t already covered?

## Appendix 3 – Support Workers & Assistant Practitioners Focus Group Topic Guide

**Imaging S&A workers – Focus group topic guide (version 3)**

**Aims of the Focus Group:**

To assess and explore:

1. Their perceptions of their role(s) within the imaging team
2. Their recruitment as a support worker
3. Comparison of roles across the imaging teams including scope of practice, autonomy in decision making, delegation, supervision
4. Training undertaken and perceived training needs
5. Opportunities for career progression including barriers and facilitators to this
6. Perspectives on the current and future role of the S&A workforce in imaging services

Where Trusts employ Bands 2/3 and Band 4 – will require separate Focus Groups.

**Focus Group schedule**

Main FG question items numbered. Potential prompts lettered underneath each main question

Introductions.

Address any queries about the study.

Clarify ‘rules’: All viewpoints are valid, everyone has the opportunity to contribute/speak, avoid referring to names specifically, clarify that if names are inadvertently used then transcripts will be redacted to ensure anonymity.

At start of recording: This is the focus group with <SW or APs> at <site code>. State number of participants and summarise their roles (e.g. 5 Band 2 , 3 Band 3, representing General, CT, MR Breast imaging)

1. Can we start by you, *very briefly*, talking about your role as a Support Worker/Assistant Practitioner here. How would you describe your general role(s) within the imaging team here.
   1. What does your working week look like? (9-5? 24/7? etc.)
   2. What is the *extent*/*scope* of role?
   3. What are your specific responsibilities?
   4. Where do you see the key differences in roles *across modalities* for support workers (band 2/3) and APs (band 4).
   5. Where do you see the key differences between levels (Bands 2, 3 and 4)
   6. What challenges do you face in your role?
2. Tell us about the ways in which you are supervised and supported in your role?
   1. Are there differences across different areas/modalities?
   2. To what extent are you given autonomy?
   3. Have there been any examples of where there has been role creep and/or where you have acted without the expected supervision? NB No attempt to highlight something wrong or trip you up in any way.
3. How have roles changed during your time here?
4. How were you recruited?
   1. Local/national?
   2. Length of employment?
5. What education and training have you undertaken and at what point? What further E&T opportunities are available to you?
   1. What are your perceived training needs?
6. Where do you see opportunities for career progression?
   1. What are the barriers and facilitators to these opportunities for career progression?
7. How do you think the role of the support worker and/or assistant practitioner in imaging services will develop moving into the future?
   1. Are there any specific barriers that you perceive in relation to these developments?
8. Is there anything else you’d like to convey about any aspect of your roles that we haven’t already covered?

If any SW or APs have been identified as having a ‘lead’ role then ask if we may follow them up for a short online interview.
